# Hyperkalaemic paralysis presenting as ST-elevation myocardial infarction: a case report

**DOI:** 10.1186/1757-1626-1-232

**Published:** 2008-10-10

**Authors:** Suriya Jayawardena, Olga Burzyantseva, Sanjay Shetty, Selvanayagam Niranjan, Ashoke Khanna

**Affiliations:** 1 Coney Island Hospital/Maimonides Medical Center, 2601 Ocean Parkway, Brooklyn, NY 11235, USA

## Abstract

**Background:**

Hyperkalaemic paralysis due to renal failure is a rare but potentially life threatening event.

**Case presentation:**

We present a patient who had sudden onset ascending flaccid paralysis. The EMS first diagnosis was acute ST-elevation myocardial infarction based on an EKG. In the emergency room (ER) due to careful history taking, serum electrolytes and repeat EKG a correct diagnosis was made and both hyperkalemia and paralysis were treated on time.

**Conclusion:**

Hyperkalaemic paralysis is rare. One must keep it in the back of the mind especially in the case of renal failure patients to avoid misdiagnosing a rapidly fatal but yet completely reversible condition.

## Background

Hyperkalaemia is seen commonly in patients with renal failure, especially in patients who are receiving hemodialysis. The EKG changes seen could be typical for hyperkalaemia or could be normal. There is no correlation of the EKG changes to the level of serum potassium levels. But on the other hand flaccid paralysis is rarely seen with hyperkaleamia.

## Case report

A 61-year-old Caucasian male was brought to the ER with an initial diagnosis of acute ST-elevation myocardial infraction by the emergency medical services (EMS). On further questioning, the patient denied history of chest pain, but complained of sudden onset generalized weakness and numbness. The patient had developed weakness of his legs and difficulty in passing urine one hour before arriving in the ER. Patient denied recent infections, headaches, nausea, vomiting, trauma or a similar neurological episode before. The patient had end stage renal failure due to poorly controlled diabetes and hypertensive heart disease for many years. He had been on routine hemodialysis thrice a week with each dialyzing session lasting for four hours for the past three years. The last dialysis was done the day prior to presentation. His medications included metoprolol 100 mg twice a day, simvastatin 40 mg daily, aspirin 81 mg daily and 70/30 insulin 30 units subcutaneously twice a day.

On examination, the patient's had a temperature of 98.2°F, respiratory rate of 20 per minute, heart rate of 90 beats per minute and a blood pressure of 150/90 mmHg. Examination of the cardiovascular system showed normal heart sounds with no murmurs. The respiratory system and gastrointestinal systems were normal. Central nervous system examination revealed an alert, awake and oriented patient with normal cranial nerve function. He had symmetrical and equal weakness of the lower limbs more than in the upper limbs. The muscle tone and the reflexes were also weaker in the lower limbs compared to the upper limbs. There was no sensory deficit. In the ER, blood tests including chemistry and complete blood count were sent. A bed side EKG and a chest X-ray were performed.

**Figure 1:**
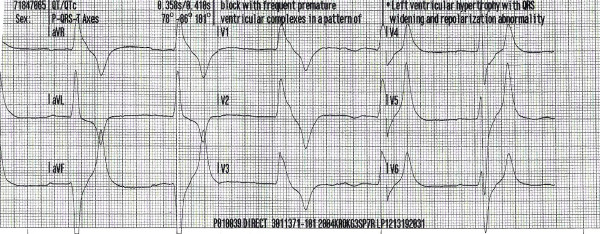
Sinus bradycardia with a 1^st ^degree heart block, wide QRS complex with hyper acute (tall) T waves – done by the EMS.

**Figure 2:**
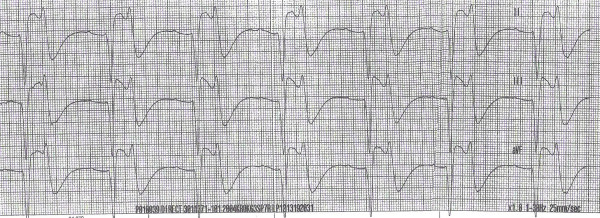
Sinus bradycardia with 1^st ^degree heart block with ST elevation in II/III and aVF (pseudo infarction pattern in the inferior leads) – done by the EMS.

**Figure 3:**
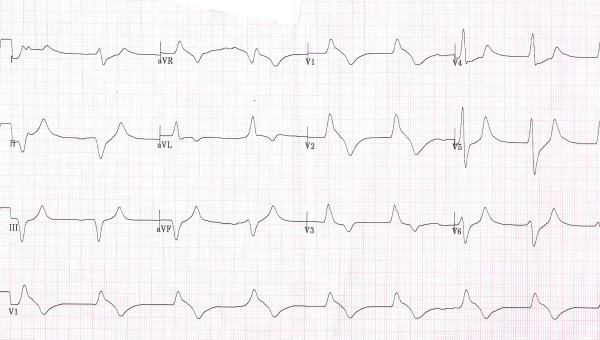
12 lead EKG showing junctional bradycardia with a heart rate of 49/min, wide QRS complex and tall T waves-done in the emergency room.

**Figure 4:**
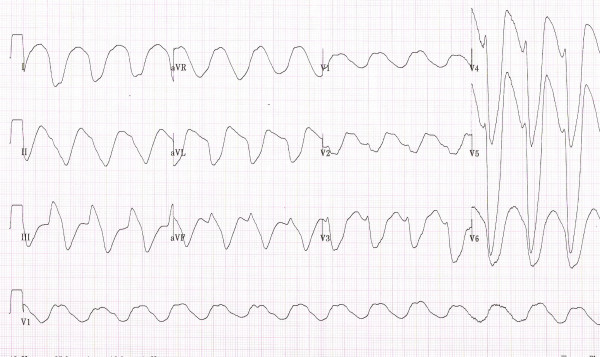
12 lead EKG showing sine-wave – done in the emergency room.

Based on the initial two EKG strips done by the EMS and the subsequent two EKGs done in the ER (Figure: 1, 2, 3, 4) and the clinical back ground of end stage renal failure the diagnosis of hyperkalaemic flaccid paralysis was made. The patient was given 10 ml of 10% calcium gluconate intravenously followed by 10 units of regular insulin, and 50 ml of 50% dextrose. The patient also received 30 ml of sodium polystyrene sulfonate (Kayexalate) orally that was repeated every hour till the patient developed a diarrhea.

**Figure 5:**
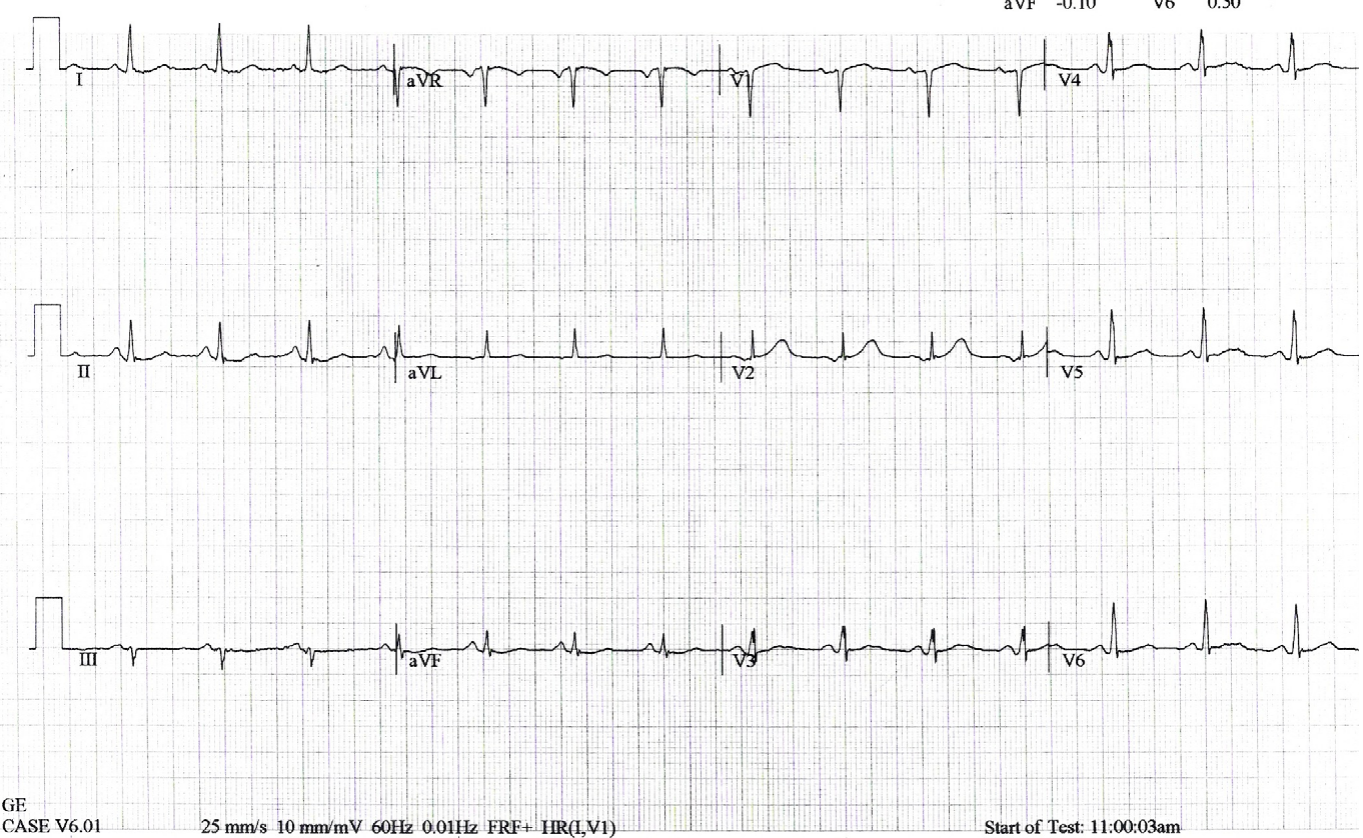
12 lead EKG done after treatment of hyperkalemia showing sinus rhythm with a heart of 88/min and complete reversal of the T wave and ST segment.

Subsequently the patient's blood results showed potassium of 6.2 mEq/L, blood urea nitrogen (BUN) of 152 mg/dl and creatinine level of 8.50 mg/dl. Patient was rushed for emergency dialysis. After three hours of dialysis, the patient recovered completely from his weakness and was able to walk. His post dialysis potassium level was 4.0 mEq/L, BUN was 47 mg/dl and creatinine was 3.57 mg/dl. The EKG had reversed back to sinus rhythm, heart rate of 88 per minute, normal T waves and ST segment (Figure 5).

## Discussion

Secondary hyperkalaemic paralysis is a rare disease entity with a rapidly fatal outcome, if not recognized and treated early. The first incident was reported in 1953 [1]. In the recent past there has hardly been any case reported. If a proper history is lacking and an EKG is not done in a timely fashion, the ascending type of flaccid paralysis with no sensory deficit may be mistaken for Guillain-Barre syndrome [2]. The exact mechanism of secondary hyperkalaemic paralysis is not clear. It is thought to be due to potassium directly influencing the muscle fiber and the cell membrane [3,4]. This is similar to the mechanism of familial hyperkalaemic paralysis, which hyperkalemia is periodic in nature. Other studies suggest a functional peripheral neuropathy [2,5].

As in our patient, hyperkalaemic paralysis is typically flaccid with intact sphincter tone and preserved sensory and cranial nerves [6]. EKG could show changes ranging from normal to tall T-waves with wide QRS, pseudo infracts, to the other extreme of having sine – wave and asystole [7-9]. EKG changes have no correlation to the level of serum potassium [10].

Management of hyperkalemia includes monitoring vital signs, repeated EKGs, assessment of serum potassium levels, hemodynamic and ventilator support if needed. Therapeutic options for treating hyperkalemia are calcium infusion, insulin and dextrose infusion, Sodium polystyrene sulfonate (Kayexalate) resins orally or suppository and dialysis therapy.

The prevention of recurrent hyperkalaemic episode should be prevented by advising the patient to avoid high potassium containing food and medications, along with routine blood testing and dialysis.

## Conclusion

Hyperkalaemic paralysis is a rare but fatal condition. The corner stone of diagnosis is a high degree of suspicion incorporated with proper clinical acumen.

## List of Abbreviations

BUN: Blood urea nitrogen; EKG: Electrocardiogram; ER: Emergency room; EMS: Emergency medical services.

## Competing interests

The above case report was written at Coney Island Hospital. The above mentioned authors have no affiliation to any other institute other than Coney Island Hospital.

## Authors' contributions

SJ and OB treated the patient and were responsible for writing the paper and looking up the back ground references. SS was responsible for proof reading and cross checking the references. SN and AK were responsible for over all coordination and final proof reading. All the above mentioned authors read and approved the final manuscript.

## Consent

A written informed consent was obtained from the patient for publication of this case report and accompanying images. A copy of the written consent will be made available on request.
